# The Microplastic–Gut Microbiota Axis: A Unifying Framework Linking Environmental Pollution to Undernutrition, Obesity, and Metabolic Dysfunction

**DOI:** 10.1016/j.cdnut.2026.109445

**Published:** 2026-07-21

**Authors:** Haiying Liu, Xiaoqing You, Yanghua Peng, Guo Wei, Wei Kong, Congfu Huang

**Affiliations:** 1 Department of Pediatrics, Longgang District Maternity and Child Healthcare Hospital of Shenzhen City (Affiliated Shenzhen Women and Children's Hospital (Longgang) of Shantou University Medical College), Shenzhen, Guangdong Province, China; 2 Department of Pediatrics, Fuzhou First General Hospital Affiliated with Fujian Medical University, Fuzhou, Fujian, China; 3 Department of Pediatrics, Shenzhen Maternity and Child Healthcare Hospital, Women and Children's Medical Center, Southern Medical University, Shenzhen, Guangdong Province, China

**Keywords:** microplastics, gut microbiota, malnutrition, short-chain fatty acids, gut barrier, metabolic endotoxemia, Western-style diets, early-life exposure, planetary health, environmental health

## Abstract

The ubiquity of plastic pollution has rendered dietary microplastic (MP) ingestion unavoidable, introducing an unrecognized yet pervasive threat to human nutritional health. This review systematically synthesizes evidence from rodent and aquatic animal models, in vitro human gut microbiota simulations, and cross-sectional human observational studies to conceptualize the “MP–gut microbiota–nutrition axis” as a unifying mechanistic framework. Our synthesis delineates 4 interconnected pathways triggered by MP-induced gut dysbiosis: *1*) disrupted energy harvest via short-chain fatty acid depletion and bile acid dysregulation; *2*) compromised gut barrier integrity leading to metabolic endotoxemia; *3*) impaired micronutrient bioavailability; and 4) appetite dysregulation through the gut–brain axis. These hazardous effects are amplified by Western-style diets and early-life exposure, positioning MP as a direct environmental determinant linking pollution to the triple burden of malnutrition. By critically evaluating dose relevance across >75 studies published between 2020 and 2025, this review rejects the notion of plastic pollution as a distant ecological concern and reframes it as a proximate, modifiable driver of global malnutrition requiring urgent integration into public health and policy.

## Graphical abstract

**Figure:**
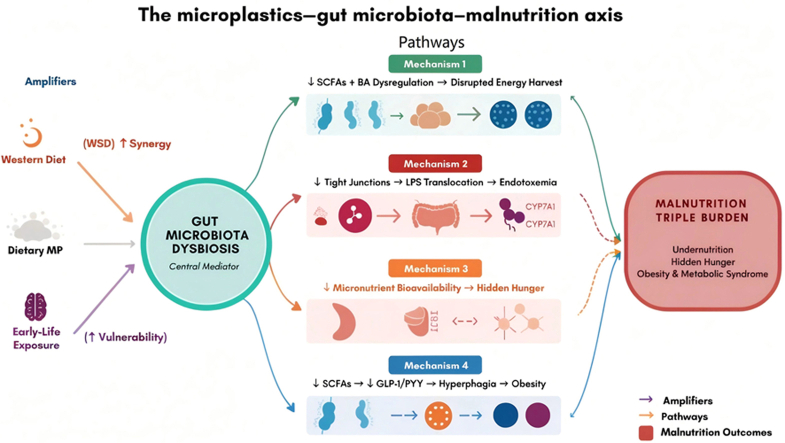

## Introduction

The 21st century has been termed the “Plastic Age” due to exponential growth in global plastic production, resulting in pervasive environmental contamination [1]. A critical consequence is the widespread distribution of microplastics (MPs; <5 mm)—synthetic polymer particles now detected across ecosystems, from oceans and soils to the atmosphere, and throughout the human food chain [2,3]. Alarmingly, MPs have been identified in human placenta, blood, liver, and gallstones [2,4], underscoring their systemic infiltration and raising urgent concerns about their health impacts. These detections have been made across diverse biospecimens—including placental tissue, whole blood, liver parenchyma, and gallstone matrices—while other human studies have quantified MP in feces and colon biopsies as markers of dietary exposure [[5], [6], [7]]. This multimatrix evidence confirms that ingested MP not only transit through the gut but can cross biological barriers to reach systemic circulation and accumulate in distant organs. Dietary intake constitutes the primary route of human exposure, positioning the gut as the first and most extensively exposed organ. This review synthesizes key advances over the past 5 y (2020–2025) in understanding MP-induced gut dysbiosis and its nutritional consequences, with emphasis on studies employing environmentally relevant exposure models and causal inference approaches [e.g., fecal microbiota transplantation (FMT)].

Concurrently, malnutrition persists as a formidable global health challenge, with its definition now expanded beyond undernutrition to encompass the “double” or “triple” burden, including micronutrient deficiencies (“hidden hunger”), obesity, and metabolic syndrome [[8], [9], [10]]. According to the latest FAO report, over 2.8 billion people could not afford a healthy diet in 2022, highlighting the substantial scale of this crisis [11]. Although traditional risk factors such as energy-dense, nutrient-poor diets are well established, they fail to fully explain the persistent and often overlapping prevalence of these malnutrition forms across diverse populations [12,13]. This etiological discrepancy raises a pressing question: could MPs, as ubiquitous environmental contaminants inadvertently consumed with every meal, represent an unrecognized environmental determinant operating through nontraditional pathways to disrupt nutritional health?

The gastrointestinal ecosystem is particularly vulnerable to such contaminants because of its direct and continuous exposure to dietary components. Often termed the host’s “second genome,” the gut microbiota play a fundamental role in maintaining nutritional health through multifaceted functions [14]. These include fermenting dietary fiber to produce short-chain fatty acids (SCFAs), supplying energy substrates, maintaining intestinal epithelial integrity, synthesizing vitamins, modulating bile acid (BA) metabolism, and supporting immune function, which are collectively critical for optimal nutrient utilization [[14], [15], [16], [17]]. Consequently, gut dysbiosis is intimately linked with various nutrition-related metabolic disorders, including obesity, undernutrition, and micronutrient deficiencies [[17], [18], [19]].

Emerging evidence from aquatic and rodent models indicates that MP exposure consistently perturbs gut microbial ecology, inducing dysbiosis characterized by reduced α-diversity, depletion of SCFA-producing taxa (e.g., Lachnospiraceae and Ruminococcaceae), and expansion of potential pathobionts [[19], [20], [21], [22], [23], [24]]. These microbial alterations are accompanied by metabolic perturbations, including disrupted energy homeostasis, impaired lipid metabolism, and systemic low-grade inflammation [21,25,26]. However, a critical caveat warrants attention: the MP concentrations employed in many experimental studies (frequently milligrams per kilograms per day) often exceed estimated human environmental exposure levels (typically micrograms per kilograms per day) [3,5]. This dose discrepancy raises fundamental questions about the translatability of experimental findings to real-world scenarios, a key consideration for environmental risk assessment. Compounding this dose discrepancy, the majority of experimental studies employ pristine, uniformly spherical particles that fail to recapitulate the irregular morphology, surface oxidation, microbial biofilm colonization, and adsorbed chemical cargo characteristic of environmentally weathered MPs [27,28]. This physicochemical divergence likely leads to systematic underestimation or mischaracterization of gut-level hazards.

To address this, we systematically synthesized existing evidence to evaluate the “MP–gut microbiota–nutrition” axis as a fundamental link between plastic pollution and malnutrition pathophysiology. We delineated 4 interconnected mechanistic pathways through which MPs, via microbiota disruption, contribute to nutritional imbalances: *1*) compromised energy and macronutrient metabolism via SCFA deficit and dysregulated BA signaling; *2*) gut barrier dysfunction and metabolic endotoxemia; *3*) impaired micronutrient bioavailability; and *4*) appetite dysregulation via the gut–brain axis. Furthermore, we examined how synergistic factors such as Western-style diet (WSD) and early-life exposure amplify these effects, positioning MPs as an emerging environmental determinant of metabolic health. Furthermore, we moved beyond a mere summary of isolated effects—such as a decline in SCFA-producing taxa or increased gut permeability—to forge a single, cohesive model: the “MP–gut microbiota–nutrition axis.” This review is the first to establish a unified framework that positions the gut microbiota as the obligatory central mediator linking environmental MP exposure to the full spectrum of human malnutrition. By connecting 4 previously disparate mechanistic pathways under 1 conceptual umbrella, this work provides a foundational hypothesis that identifies critical knowledge gaps and charts a targeted course for future translational research. Ultimately, it issues a call for a necessary paradigm shift: to reframe plastic pollution not as an insidious but distant ecological issue but as a direct, modifiable, and pervasive threat to human nutritional health.

Beyond its conceptual novelty, the MP–gut microbiota–nutrition axis carries immediate implications for nutritional science. It challenges the prevailing view that malnutrition arises solely from dietary inadequacy or excess by identifying an environmental pollutant that independently reprograms host nutrient utilization through the microbiome. This framework recasts plastic pollution as a modifiable nutritional risk factor rather than a distant ecological concern into a modifiable nutritional risk factor, and it suggests that dietary interventions alone may be incompletely effective in populations with high MP exposure, because the microbiota-mediated machinery for nutrient extraction, barrier integrity, and appetite regulation has been fundamentally altered. The following sections systematically evaluate this hypothesis across 4 interconnected domains of nutritional homeostasis.

## Methods

For this review, we conducted a comprehensive literature search in PubMed, Web of Science, and Scopus from inception through 31 October, 2025. The search strategy combined terms related to MPs (e.g., “microplastic,” “nanoplastic”), gut microbiota (e.g., “gut microbio,” “intestinal microbio,” “dysbiosis”), and malnutrition outcomes (e.g., “malnutrition,” “obesity,” “undernutrition,” “micronutrient deficiency,” “metabolic syndrome”).

We included original studies investigating oral exposure to MPs or nanoplastics that reported outcomes related to gut microbiota and/or nutritional status. Given the conceptual nature of this review and the substantial heterogeneity in exposure parameters (polymer types, sizes, doses, and durations) and outcome measures across the included studies, a quantitative meta-analysis was not feasible. Instead, we employed a narrative synthesis approach, categorizing evidence according to key mechanistic themes: energy metabolism, barrier function, micronutrient bioavailability, and gut–brain axis signaling. This analytical strategy enabled the construction of a unified mechanistic framework for the microplastic–gut microbiota–nutrition axis.

The literature search and screening process were informed by PRISMA guidelines to ensure transparency. A PRISMA flow diagram detailing the identification, screening, and inclusion of studies is provided in Supplementary Figure 1. This review was not registered in PROSPERO as it is a narrative synthesis rather than a systematic review. A summary of included study characteristics is presented in Supplementary Table 1.

## The First Encounter: Fate and Interactions of Microplastics With the Gut Microbiota in the Gastrointestinal Tract

**FIGURE 1:**
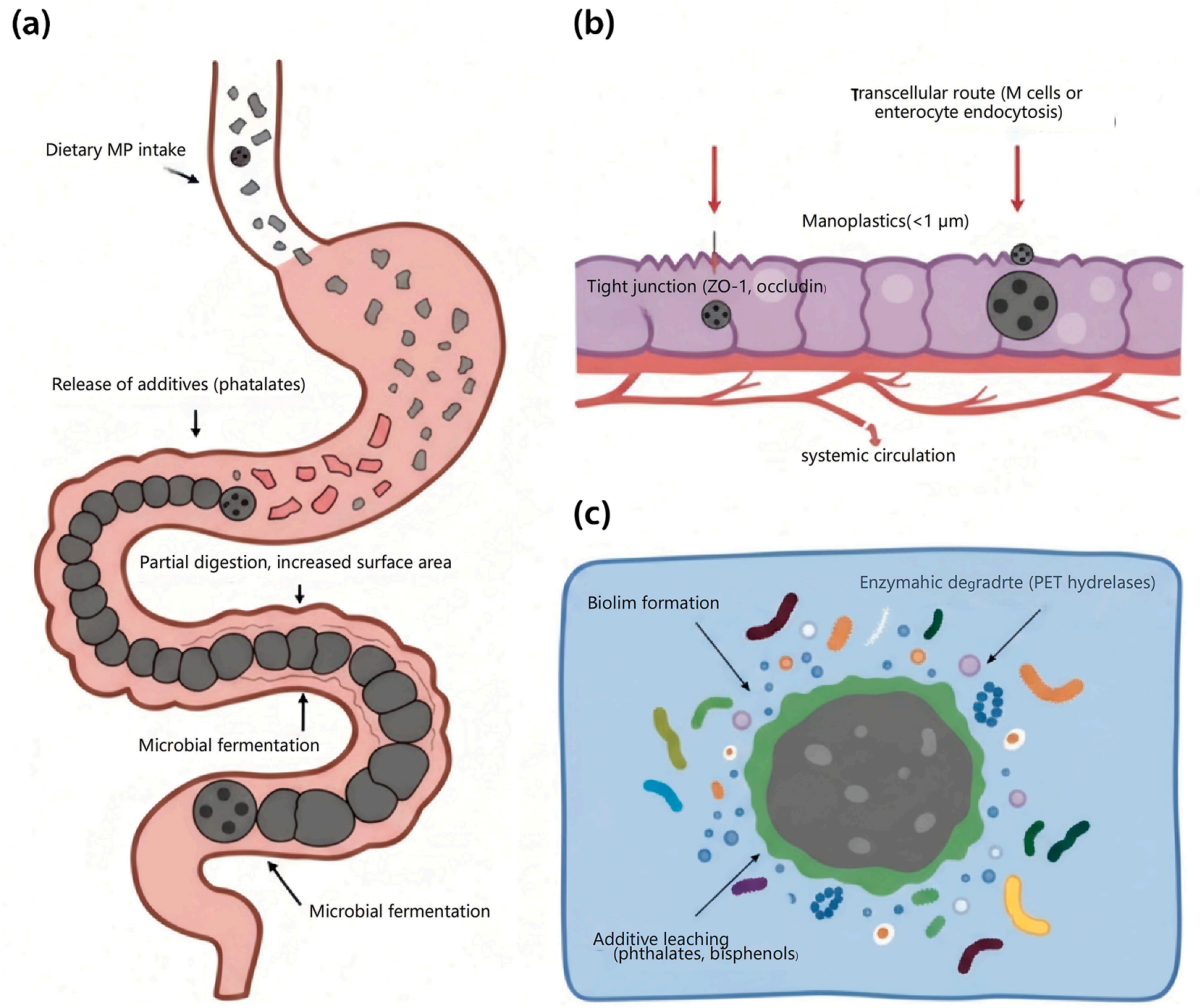
Gastrointestinal fate and biotransformation of MPs. Schematic representation of the journey and interactions of ingested MP in the human gastrointestinal tract. (A) After ingestion, MPs traverse the gastrointestinal tract, with their physicochemical properties (size, shape, polymer type, surface charge) influencing retention time, bioaccessibility, and toxic potential. (B) Nanosized MP (<1 μm) may translocate across the intestinal epithelium via paracellular routes (through compromised tight junctions) or transcellular routes (via M cells or enterocyte endocytosis), potentially reaching systemic circulation. (C) In the colon, MPs interact extensively with the gut microbiota through processes including biofilm formation on MP surfaces, microbial enzymatic degradation (e.g., via polyethylene terephthalate hydrolases identified in human gut microbiota), and leaching of additives (e.g., phthalates, bisphenols). These bidirectional interactions collectively alter MP physicochemical properties and toxicity potential, while also affecting microbial community structure and function. MP, microplastic.

The initial interaction between ingested MP and the complex gut ecosystem is a critical starting point that triggers subsequent physiological effects. Understanding this “first encounter” is essential for elucidating the entire “MP–gut microbiota–nutrition” axis. The gastrointestinal journey and initial interactions of MP are summarized in Figure 1.

Our systematic synthesis of the extant literature reveals that MP exposure consistently disrupts gut microbial ecology across diverse models. Key reproducible patterns include a marked reduction in microbial α-diversity, significant shifts in β-diversity [20,22,29], a decreased abundance of pivotal SCFA-producing taxa (e.g., families Lachnospiraceae and Ruminococcaceae) [20,23,30], and a concomitant enrichment of potential pathobionts [23,31]. These dysbiotic patterns set the stage for the multifaceted nutritional impairments detailed in the following sections, which we have integrated into a conceptual framework to illustrate the overarching “MP–gut microbiota–nutrition” axis.

### The gastrointestinal journey of MPs

Dietary intake is the primary route of human exposure to MP, through contaminated seafood, salt, drinking water, honey, and food packaging materials [2,32]. The physicochemical properties of MP—including size, shape, polymer type, and additive content—collectively determine their gastrointestinal fate and toxic potential [1,3,5]. For instance, smaller nanoplastics exhibit greater potential to cross the intestinal barrier into systemic circulation compared with larger microparticles [33], whereas fibrous MPs may cause distinct physical irritation to gut tissues [34]. In vitro simulations of human digestion indicate that the properties of MP influence their fate during digestion and may involve the release of additives, such as phthalates [35]. A study on disposable plastic tableware further highlights the reality of oral exposure, showing that thermal exposure can release MP and alter the human gut microbiota and metabolome [36]. The physicochemical properties of MP not only determine their gastrointestinal fate but also govern their hazard potential. Smaller nanoplastics (<1 μm) exhibit enhanced capacity to translocate across the intestinal epithelium [33], potentially delivering these hazardous particles to systemic circulation and distant organs, a pathway implicated in multiorgan toxicity observed in murine models [20,34]. Furthermore, the leaching of additive chemicals (e.g., phthalates, bisphenols) from ingested MP [35] represents an additional hazard dimension, as these compounds are recognized endocrine disruptors with documented metabolic toxicity [37]. The gastrointestinal tract therefore represents both the primary exposure route and the initial site of biological injury induced by MP.

### Microbial “biotransformation” of microplastics

The gut microbiota are not a passive target but actively interact with MP. Research has identified insect larvae (e.g., mealworms, wax moths) whose gut microbes can degrade polymers like polyethylene (PE) and polystyrene (PS) [[38], [39], [40]]. More importantly, hydrolytic enzymes capable of degrading polyethylene terephthalate (PET) have been identified in the human gut microbiota [41], and in vitro simulations provide the first evidence of plausible PET biodegradation during human digestion [42]. This biotransformation may involve biofilm formation on MP surfaces [43,44], altering their surface properties, adsorption capacity, and additive release kinetics, thereby modulating their toxicity (Figure 1) [37,45]. For example, plasticizers leached from MPs can inhibit microbial metabolic activity [37]. Whether such biodegradation occurs at physiologically relevant rates in the human gut, and whether it attenuates or amplifies MP toxicity, remains an open question with implications for risk assessment and bioremediation strategies. Thus, microbial biotransformation of MP is a dual-faceted process, potentially mitigating MP persistence but also possibly enhancing toxicity through physicochemical modifications, as summarized in Figure 1. Although the identification of hydrolytic enzymes such as PET hydrolases in the human gut microbiota suggests a capacity for MP biodegradation, the physiological relevance of this process in vivo requires further investigation. Future studies should assess the metabolic outcomes of such biotransformation, specifically whether it mitigates MP toxicity or generates bioactive intermediates that further disrupt gut homeostasis.

### MPs as a microbiota stressor

Beyond potential biodegradation, MPs primarily act as stressors that disrupt microbial homeostasis. A substantial body of evidence demonstrates that MP exposure directly induces gut dysbiosis, characterized by several reproducible patterns across animal models and human-relevant systems.

First, regarding community structure, a meta-analysis in aquatic species indicated that MP predominantly alter β-diversity rather than simply reducing α-diversity [29]. For instance, exposure to PS MP in mouse models has been shown to significantly decrease α-diversity and alter β-diversity [20,22].

Second, specific taxonomic shifts are commonly observed, including alterations in the Firmicutes-to-Bacteroidetes ratio, a change frequently associated with metabolic abnormalities in MP-exposed models [22,30] and widely recognized as a hallmark of obesity-related gut dysbiosis in human metabolic disorders [46]. Crucially, many studies report a significant decrease in beneficial SCFA-producing bacteria, such as those within the families Lachnospiraceae and Ruminococcaceae [20,23,30], alongside an increase in potentially pathogenic or opportunistic bacteria, including certain genera within the Proteobacteria phylum [23,31].

Third, evidence from human-relevant models further supports these findings. In vitro simulations utilizing human gut microbiota communities have confirmed that MP, including PET and polylactic acid (PLA), can specifically alter bacterial composition and function, providing direct evidence of MP-induced disruption in a human context [42].

Collectively, these findings establish MP as a potent environmental stressor capable of disrupting gut microbial homeostasis. The consistency of these dysbiotic patterns across species suggests conserved mechanisms with relevance to human health. Building on this foundation, the next section examines how MP-induced microbial disruption translates into specific nutritional impairments.

### Unraveling the Mechanisms: How the Microplastic–Microbiota Axis Disrupts Nutritional Status

**FIGURE 2:**
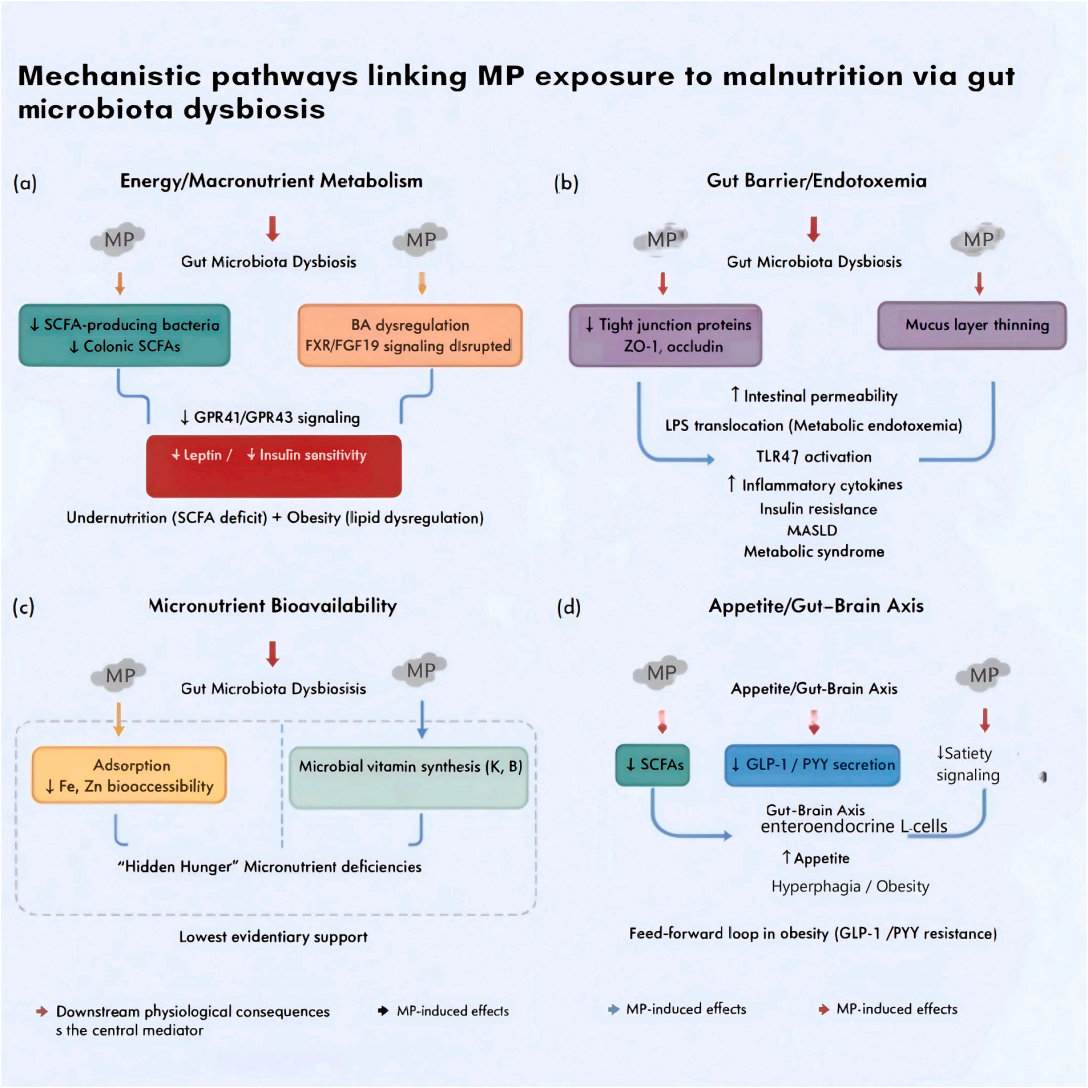
Hazard pathways linking MP-induced gut dysbiosis to nutritional impairments. The figure illustrates 4 interconnected toxicity pathways through which MP, via gut microbiota disruption, exert their hazardous effects on host nutritional status. Each panel (A–D) depicts a distinct mechanism of MP-induced harm, corresponding to the pathways detailed in section “Unraveling the Mechanisms: How the Microplastic–Microbiota Axis Disrupts Nutritional Status.” (A) Disruption of energy and macronutrient metabolism. MP depletes SCFA-producing bacteria (e.g., Lachnospiraceae, Ruminococcaceae), reducing colonic SCFA concentrations and altering BA metabolism via FXR-FGF19 signaling, thereby compromising lipid digestion and absorption. (B) Gut barrier dysfunction and metabolic endotoxemia. MP downregulate tight junction proteins (e.g., ZO-1, occludin), increasing intestinal permeability and facilitating the translocation of bacterial lipopolysaccharide into circulation. This induces chronic low-grade inflammation (metabolic endotoxemia), promoting insulin resistance and metabolic dysfunction. (C) Impaired micronutrient bioavailability. MP may adsorb essential trace elements (e.g., iron, zinc) in the gut lumen, reducing their bioaccessibility. Concurrently, MP-induced dysbiosis may disrupt microbial synthesis of vitamins (e.g., vitamin K, B vitamins), potentially contributing to micronutrient deficiencies. (D) Dysregulation of appetite via the gut–brain axis. MPs reduce SCFA-producing bacteria, impairing SCFA-mediated secretion of anorexigenic hormones (glucagon-like peptide-1, GLP-1; peptide YY, PYY) from enteroendocrine cells, leading to aberrant central appetite control and increased energy intake. Red arrows indicate MP-induced effects; blue arrows indicate downstream physiological consequences. This integrative model synthesizes evidence from preclinical studies; the quantitative contribution of each pathway to human malnutrition requires validation using environmentally relevant exposure models. BA, bile acid; B vitamins, B-group vitamins; FXR, farnesoid X receptor; FGF19, fibroblast growth factor 19; GLP-1, glucagon-like peptide-1; MP, microplastic; PYY, peptide YY; SCFA, short-chain fatty acid; ZO-1, zonula occludens-1.

Upon ingestion, MPs interact extensively with the gut microbial community, triggering a cascade of events that compromise host nutritional homeostasis. This section systematically delineates the key molecular and physiological pathways through which MPs impair nutrient metabolism by perturbing the gut microbiota, with a focus on causal evidence and specific malnutrition outcomes. In the following sections, we dissect these pathways in detail, with an integrative overview provided in Figure 2.

To decipher the pathways linking MP-induced dysbiosis to nutritional deficits, we synthesized evidence into a cohesive mechanistic model (Figure 2). This synthesis integrates preclinical and in vitro findings to posit 4 interconnected pathways: disrupted energy harvest, gut barrier dysfunction, impaired micronutrient bioavailability, and appetite dysregulation via the gut–brain axis. Each pathway is detailed below.

Of these 4 pathways, 2 (disrupted energy metabolism, barrier dysfunction) are supported by convergent preclinical evidence and have been causally validated through FMT studies [22,47,48]. The micronutrient bioavailability pathway remains mechanistically plausible but awaits in vivo confirmation, whereas the appetite dysregulation pathway has initial behavioral evidence in rodents that requires human translation. Below, we dissect each pathway in detail.

### MP-induced dysbiosis disrupts host energy harvest, contributing to obesity and undernutrition

MP-induced gut dysbiosis acts as a key pathogenic driver that reconfigures host energy harvest and macronutrient utilization, primarily through the impairment of SCFA metabolism and the disruption of BA signaling (Figure 2A). These alterations collectively promote metabolic dysfunction, contributing to both obesity and undernutrition.

A central mechanism involves the depletion of microbially derived SCFAs, primarily acetate, propionate, and butyrate, which serve as crucial energy substrates and metabolic regulators [14]. Exposure to PS or PE MP in mice and zebrafish significantly reduces the abundance of key SCFA-producing bacteria (e.g., families Lachnospiraceae and Ruminococcaceae), leading to decreased colonic SCFA concentrations [20,22,23,30]. This decline not only reduces the caloric yield from dietary fiber but also attenuates SCFA-mediated signaling via G-protein–coupled receptors (GPR41/GPR43), which normally promotes leptin secretion and insulin sensitivity. Hence, the energy deficit is compounded by a loss of metabolic-regulatory signals, creating a dual mechanism for both undernutrition and metabolic inflexibility. MPs also alter the microbial repertoire of carbohydrate-active enzymes (CAZymes), potentially limiting the breakdown of complex dietary polysaccharides [40].

Concurrently, MPs interfere with BA metabolism, which is critically modulated by gut microbes. MP-induced dysbiosis reshapes the BA pool, disrupting the farnesoid X receptor (FXR) and fibroblast growth factor 19 (FGF19) signaling pathways, thereby impairing lipid emulsification, digestion, and absorption [41,42]. Co-exposure studies, such as those combining MP with tributyltin, demonstrate exacerbated BA dysregulation and metabolic dysfunction in mice [31]. Importantly, FMT from MP-exposed mice to germ-free recipients recapitulates metabolic defects, providing causal evidence that microbiota disruption is necessary for MP-induced impairments in energy homeostasis [47]. Such causal validation is essential for establishing MP as a direct etiological agent in metabolic dysfunction, moving beyond correlative observations.

These mechanistic insights derive predominantly from rodent and aquatic models. A critical consideration in extrapolating these findings to human health is the substantial discrepancy between experimental exposure doses and estimated environmental intake levels. The MP doses employed in rodent studies demonstrating metabolic disruption (frequently 0.1–100 mg/kg/d) [20,22,41] are 10^3^- to 10^6^-fold higher than estimated human daily intake (approximately 0.1–5 μg/kg/d via diet) [3,5]. For context, human MP intake via drinking water has been estimated at 0.06 mg/kg/d, whereas rodent studies have used doses as high as 100–2000 mg/kg/d—a discrepancy of over 30,000-fold. A systematic review of MP dose-responses highlighted that many adverse effects observed at high doses may not occur at environmentally relevant concentrations, with threshold and hormetic responses frequently documented [49].

Encouragingly, a growing number of studies have begun to employ environmentally relevant doses in rodent models. One study calculated that a human MP intake of ∼0.2–0.6 mg/kg body weight translates to a mouse equivalent dose of 2.5–7.4 mg/kg body weight [50]. At these lower doses, significant biological effects—including altered gut microbiota composition, decreased ATP concentrations, and disrupted lipid metabolism—have still been detected, suggesting that MP toxicity may not be strictly dose-proportional. Nevertheless, the majority of studies demonstrating the full spectrum of metabolic and barrier-disrupting effects reviewed herein have used doses substantially above estimated human exposure levels, a limitation that must be acknowledged when interpreting the mechanistic framework.

The dose–response relationship for MP-induced effects is likely nonlinear [49]. This complexity is underscored by NOAEL (no observed adverse effect level) studies in rodents: a health-based framework for California drinking water identified NOAEL values for polystyrene microspheres ranging from 0.015 to 3 mg/kg/d for various reproductive and endocrine endpoints [49]. These values often exceed estimated human exposure levels by several orders of magnitude, suggesting that classical toxicological risk assessment paradigms would currently classify dietary MP exposure as low risk. However, the NOAEL framework may not adequately capture the chronic, low-grade inflammatory and microbiome-mediated effects that are the focus of this review, as these endpoints are not routinely assessed in standard regulatory toxicity tests. Thus, although the dose discrepancy warrants caution in extrapolation, it does not invalidate the mechanistic plausibility of the pathways described; rather, it underscores the urgent need for chronic, low-dose exposure studies that specifically assess microbiome-dependent outcomes.

The causal evidence from FMT studies [47] underscores that the microbiota mediates this toxicity, highlighting the gut ecosystem as a critical therapeutic target for mitigating MP-induced metabolic harm. However, translation to human energy homeostasis requires caution because of more complex endocrine and behavioral adaptations [14]. From a nutritional perspective, this pathway bridges environmental pollution and the 2 extremes of energy imbalance. By simultaneously depleting SCFA-producing bacteria—which diminishes caloric harvest from dietary fiber—and disrupting BA signaling, which impairs lipid handling, MP exposure may concurrently elevate risks for undernutrition and obesity. This dual-risk profile mirrors the nutrition transition observed in low- and middle-income countries, where undernutrition and obesity increasingly coexist, and suggests that MP exposure may be an underappreciated environmental contributor to this epidemiological phenomenon.

### MPs compromise intestinal barrier integrity, triggering metabolic endotoxemia and systemic inflammation

The gut microbiota are essential for maintaining intestinal barrier integrity, and disruption of this protective function represents a major mechanism of MP toxicity. We summarize evidence that MPs disrupt this homeostasis, leading to a thinner mucus layer and downregulation of tight junction proteins (e.g., ZO-1, occludin) [24,36,51,52], alterations that directly impair the gut’s defensive barrier against luminal contents. For example, polyvinyl chloride MP exposure in mice significantly decreased occludin and ZO-1 expression in the colon, indicating impaired barrier function [51].

Similarly, studies in shrimp have demonstrated associations between MP colonization and intestinal microbiota alterations, further supporting the link between MP exposure and gut barrier disruption [52]. This increased intestinal permeability facilitates the translocation of bacterial lipopolysaccharide (LPS) into the circulation, inducing a state of chronic low-grade inflammation known as metabolic endotoxemia [53,54]. This inflammatory milieu promotes insulin resistance in the liver and peripheral tissues, thereby linking gut dysbiosis to systemic metabolic disturbances [26,55]. This cascade illustrates a specific pathophysiological sequence: MP-induced dysbiosis compromises tight junction integrity, enabling luminal LPS to enter the circulation and activate Toll-like receptor 4 (TLR4)-mediated inflammatory pathways in the liver and adipose tissue. However, key molecular details remain unresolved, particularly whether certain MP-associated pathobionts produce metabolites that directly regulate occludin phosphorylation, and how host genetic variants in barrier-related genes modulate susceptibility. This mechanistic gap limits our ability to identify at-risk populations. The resultant metabolic endotoxemia promotes insulin resistance and other features of the metabolic syndrome [26,55], demonstrating that MP-induced gut injury has consequences extending far beyond the gastrointestinal tract. PS MP exacerbate high-fat diet-induced metabolic dysfunction-associated steatotic liver disease (MASLD) in mice by disrupting the gut barrier [56], illustrating synergistic toxicity between MP and dietary risk factors. The causal role of microbiota is further supported by FMT experiments, in which microbiota from MP-exposed donors transferred gut barrier defects to recipient animals [48].

A critical caveat is that the evidence for MP-induced barrier dysfunction and consequent metabolic endotoxemia stems largely from controlled animal experiments, often employing MP concentrations that may exceed typical human environmental exposure (see Section “MP-induced dysbiosis disrupts host energy harvest, contributing to obesity and undernutrition” for a detailed discussion of dose discrepancies) [3]. The extrapolation of these findings to humans must account for differences in gut anatomy, baseline microbiota resilience, and the multifactorial nature of leaky gut in human populations. Whether real-world MP exposure is a significant contributor to systemic inflammation and metabolic endotoxemia in humans remains an open and pivotal question. This question is further contextualized by emerging evidence that the dose–response relationship for MP-induced barrier disruption may not be linear. Studies using human-relevant in vitro models suggest that while high-dose exposures (≥10 μg/mL) consistently impair tight junction protein expression, low-dose exposures closer to estimated human gut concentrations may induce adaptive responses or no detectable effects [57]. However, it must be noted that some human cell-based models have detected barrier alterations—such as decreased transepithelial electrical resistance (TEER) and redistribution of claudin-1—at concentrations ∼5 μg/mL. Notably, a recent in vivo study in infant mice demonstrated that even an exceedingly low ambient concentration of polystyrene MP (20 ppb, ∼0.02 μg/mL) was sufficient to inflict considerable intestinal barrier damage, as evidenced by lessened mucus secretion, elevated intestinal fatty acid-binding protein concentrations, and decreased secretory IgA concentrations [58]. Although this study utilized a high-fat diet background, which may independently influence barrier function, the low-dose effect is mechanistically instructive. These findings question the assumption of a linear no-effect threshold and instead suggest that the dose–response relationship is likely nonmonotonic, shaped by particle size, surface chemistry, host developmental stage, and the specific model system employed. Particularly during early life, when intestinal defenses are immature, the barrier-disrupting effects of MPs may emerge at far lower concentrations than previously assumed. Incorporating these sensitive developmental endpoints into future risk-assessment frameworks is therefore essential. Furthermore, the presence of co-stressors, such as WSD, which themselves compromise barrier function, may lower the threshold for MP toxicity [54,55], suggesting that vulnerable populations with pre-existing gut permeability may be at greater risk even at environmental exposure levels. Collectively, these findings establish a pathway (summarized in Figure 2B) from MP-induced dysbiosis to barrier failure, metabolic endotoxemia, and systemic metabolic dysregulation, which may contribute to obesity and metabolic syndrome under exposure conditions that impair barrier integrity; the quantitative importance of this pathway at typical human intake amounts remains to be established.

Importantly, the above findings derive predominantly from studies using pristine MP particles, which may not fully represent the physicochemical complexity of environmental MP exposure. Emerging comparative evidence indicates that environmentally aged MPs—characterized by surface oxidation, increased roughness, and biofilm colonization—may exert more pronounced intestinal toxicity than their pristine counterparts. In a subchronic dietary exposure study in mice, UV-aged polyethylene MPs caused more severe hepatic dysfunction and gut microbiota disruption than pristine particles at the same doses (0.01 and 1 mg/d), with aged particles uniquely enriching Akkermansia, Parasutterella, and Turicibacter while reducing Lactobacillus [27]. This differential dysbiosis was accompanied by elevated reactive oxygen species in the small intestine and liver, suggesting that surface oxidation products generated during environmental aging amplify oxidative stress-mediated barrier injury [27]. Notably, these dose concentrations (0.01–1 mg/d) are closer to environmentally relevant ranges than the higher doses used in many other studies [50], suggesting that aged MPs may pose a greater hazard even at lower exposure levels. Biofilm colonization of aged MP surfaces further alters their toxicity profile: biofilm-coated MPs exhibit modified adsorption capacities for environmental contaminants such as heavy metals and antibiotics, potentially serving as vectors that deliver concentrated toxicants directly to the intestinal epithelium [59].

This differential dysbiosis was accompanied by elevated reactive oxygen species in the small intestine and liver, suggesting that surface oxidation products generated during environmental aging amplify oxidative stress-mediated barrier injury. Similarly, UV-aged PS nanoplastics aggravated intestinal barrier damage through overproduction of reactive oxygen species and hydroxyl radicals in a murine model, whereas pristine particles at equivalent concentrations produced milder effects [28]. Biofilm colonization of aged MP surfaces further alters their toxicity profile: biofilm-coated MPs exhibit modified adsorption capacities for environmental contaminants such as heavy metals and antibiotics, potentially serving as vectors that deliver concentrated toxicants directly to the intestinal epithelium [59]. These findings collectively suggest that the barrier-disrupting effects documented in pristine-particle studies may represent the lower bound of true hazard potential. The nutritional significance of this barrier pathway extends beyond metabolic disease. Chronic low-grade inflammation driven by a compromised gut barrier diverts energy and micronutrients toward immune activation rather than growth and tissue maintenance—a phenomenon well characterized in environmental enteric dysfunction. Chronic MP exposure may partially recapitulate this pathophysiology, creating a state of functional nutrient malpartitioning in which dietary adequacy does not translate into nutritional sufficiency.

### MP may reduce micronutrient bioavailability, potentially exacerbating "hidden hunger"

Beyond their effects on energy metabolism, MPs may compromise nutritional status through additional hazardous mechanisms: reducing the bioavailability of essential micronutrients and disrupting microbial vitamin synthesis. Although less established than the pathways discussed above, this potential toxicity pathway warrants careful consideration given the global burden of micronutrient deficiencies.

#### Direct adsorption of micronutrients

The high surface area and charge properties of MP enable the adsorption of essential trace elements such as iron and zinc in vitro [60]. This physical interaction could theoretically reduce the pool of bioaccessible minerals within the gut lumen. However, the nutritional relevance of this phenomenon in vivo remains unproven, as in vitro models cannot replicate the dynamic processes of digestion, mucin binding, and active transport that determine micronutrient absorption.

#### Disruption of microbial vitamin synthesis

Gut microbiota contribute to the host’s vitamin supply, synthesizing compounds such as vitamin K and several B vitamins [14]. MP-induced dysbiosis, characterized by shifts in microbial community structure, could theoretically impair the metabolic capacity for vitamin biosynthesis. Although metagenomic predictions in some MP-exposed models have revealed potential alterations in vitamin metabolism pathways [17,20], direct evidence causally linking MP exposure to measurable deficits in host vitamin status is currently absent.

Summary and perspective: among the 4 pathways discussed in this review, the micronutrient bioavailability mechanism carries the lowest level of evidentiary support. These dual mechanisms, direct adsorption and impaired microbial synthesis, are mechanistically plausible but currently constitute a hypothesis requiring rigorous validation (Figure 2C). The contribution of MPs to the global burden of micronutrient deficiencies remains unknown and likely depends on coexisting factors such as baseline dietary intake, gut health, and, critically, exposure dose. In vitro adsorption studies demonstrating MP binding of trace elements typically employ concentrations (milligrams per milliliter range) far exceeding those expected in the human gut lumen [60], and evidence linking dysbiosis to impaired vitamin synthesis derives largely from high-dose animal studies [17,20]. Future investigations employing physiologically relevant MP concentrations in advanced gut models [e.g., Simulator of the Human Intestinal Microbial Ecosystem (SHIME) with integrated micronutrient absorption assessment] are urgently needed to determine whether this pathway contributes to human “hidden hunger,” particularly in populations already at risk. If validated, this mechanism would add a critical dimension to the MP hazard profile, linking plastic pollution directly to the micronutrient deficiencies affecting over 2 billion people worldwide. However, the current evidence gap necessitates cautious interpretation; establishing whether this pathway operates at environmentally relevant concentrations or represents a high-dose phenomenon with limited real-world significance is essential for accurate risk assessment and prioritization of public health interventions. A critical next step is to determine whether MPs, at concentrations modeling the human gut lumen, can measurably reduce the bioaccessibility of iron and zinc using physiologically based extraction tests coupled with SHIME models. Furthermore, stable isotope studies in animal models could definitively quantify whether MP-induced dysbiosis alters the net absorption and incorporation of these minerals into host tissues. Prospective cohort studies in populations with known micronutrient deficiencies and elevated MP exposure should also assess whether fecal MP concentration correlates with established biomarkers of iron and zinc status. For the nutrition community, even a modest reduction in micronutrient bioavailability attributable to chronic MP exposure would carry substantial public health significance, given that >2 billion people already have marginal iron or zinc status. This pathway, if validated, would reposition MP pollution as a previously unrecognized contributor to the global burden of hidden hunger—one that operates independently of dietary intake and thus requires complementary intervention strategies beyond food fortification and supplementation.

### MP disrupts gut–brain axis signaling, promoting hyperphagia and obesity risk

The microbiota–gut–brain axis represents a vulnerable target for MP toxicity; disruption of this bidirectional communication pathway can promote maladaptive eating behaviors and metabolic dysregulation. MP reduce SCFA-producing bacteria, impairing anorexigenic hormone release like glucagon-like peptide-1 (GLP-1) and peptide YY (PYY), which promote satiety [14], a mechanism that may contribute to hyperphagia and obesity risk. The reduction in SCFAs and microbial disruption caused by MPs may impair this endocrine signaling, thereby dysregulating central appetite control and energy intake [26,61]. This is consistent with the established role of SCFAs in stimulating the release of anorexigenic hormones [62]. Supporting this, PS MP exposure has been shown to alter feeding behavior in mice, potentially via gut–brain axis communication [63]. Moreover, malnutrition-induced cognitive and microglial impairments are linked to gut microbiota alterations [19], indicating that MP could indirectly affect central nervous system functions involved in appetite regulation.

Evidence for MP-induced appetite dysregulation derives predominantly from rodent studies, with MP doses that alter feeding behavior (typically 1–100 mg/kg/d) substantially exceeding estimated human exposure levels (micrograms per kilogram per day) [31,61]. Although these models provide valuable mechanistic insights, direct translation to human appetite control remains speculative, as human eating behavior is influenced by complex psychological, social, and cultural factors not captured in these models [14]. A recent zebrafish study using environmentally relevant concentrations of polyethylene and polyester MP found that although gut microbial composition shifted (altered β-diversity), α-diversity remained stable, and the microbiome demonstrated resilience to MP-induced stress [64]. This suggests that at realistic exposure levels, compensatory mechanisms may mitigate some effects observed at higher doses. Nevertheless, this resilience may be species-specific. The divergence between the zebrafish data [64] and the pronounced metabolic effects typically observed in rodents may be illuminated by a recent meta-analysis encompassing over 1300 gut microbiota samples across 6 animal categories. That study demonstrated that mice exhibit markedly heightened susceptibility to MP-induced gut dysbiosis compared with other species, including zebrafish, as reflected by greater losses in diversity and more pronounced shifts in the Firmicutes/Bacteroidetes ratio; exposure duration, rather than dose alone, emerged as the primary driver of these community-level perturbations [65]. These findings reinforce the hypothesis that species-specific microbiota architecture—particularly the higher baseline abundance of Firmicutes and Bacteroidetes in mammals—renders the murine and, potentially, the human gut more vulnerable to MP-induced metabolic interference. A comparative perspective, therefore, highlights the necessity of conducting parallel studies across phylogenetically distinct hosts to disentangle universal mechanisms from taxa-specific responses. We note further that the above findings derive exclusively from studies using pristine MP particles. Emerging evidence in zebrafish demonstrates that UV-weathered polypropylene MP [27,28] and aged PLA MP [7,59] induce more pronounced gut microbial community disruption and neurobehavioral alterations than their pristine counterparts, effects potentially attributable to increased surface roughness facilitating higher gastrointestinal retention and enhanced leaching of oxidative degradation byproducts into the gut lumen. Whether appetite-regulating pathways exhibit similar dose-dependent thresholds remains to be determined, and the contribution of this pathway to human obesity awaits confirmation in human studies. This suggests that at realistic exposure levels, compensatory mechanisms may mitigate some effects observed at higher doses. Whether appetite-regulating pathways exhibit similar dose-dependent thresholds remains to be determined, and the contribution of this pathway to human obesity awaits confirmation in human studies. This proposed mechanism of appetite dysregulation is depicted in Figure 2D. Thus, MP may disrupt the gut–brain axis to dysregulate energy intake, promoting hyperphagia and contributing to obesity, while also potentially affecting neurocognitive aspects of nutritional health.

The 4 pathways connect the MP–microbiota axis to distinct malnutrition outcomes. Mechanism 1 (Energy/Macronutrient) contributes to obesity through lipid dysregulation and to undernutrition via SCFA deficit. Mechanism 2 (Barrier/Endotoxemia) links MP exposure to obesity and metabolic syndromes through inflammation and insulin resistance. Mechanism 3 (Micronutrients) presents a mechanistically plausible driver of “hidden hunger.” Mechanism 4 (Appetite) primarily promotes obesity through amplified energy intake, though it may indirectly affect undernutrition.

**FIGURE 3:**
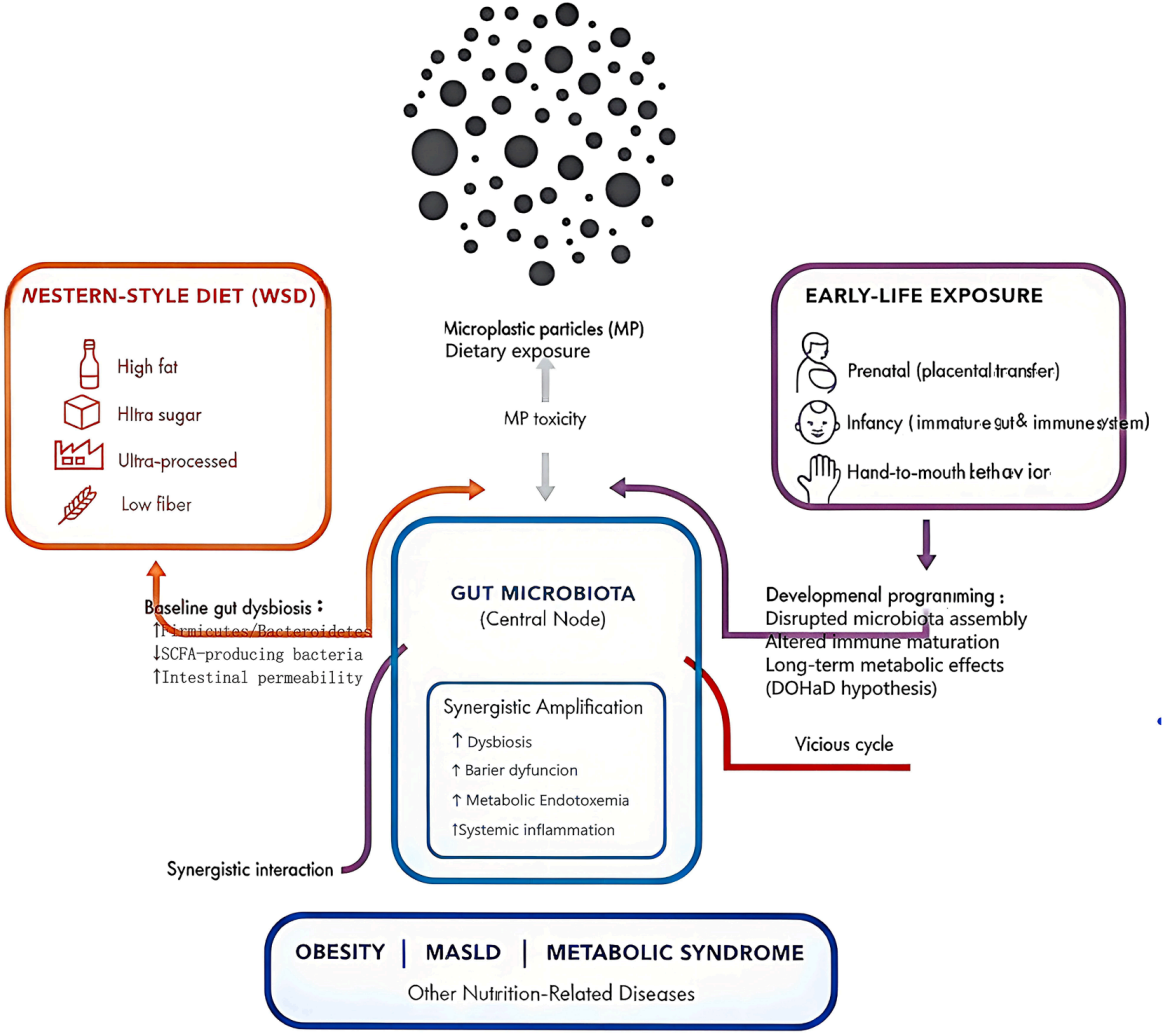
Synergistic amplifiers of MP toxicity. MPs interact synergistically with Western-style diets (characterized by high fat, sugar, and ultraprocessed foods) and early-life exposure (prenatal and infant periods) to amplify gut dysbiosis, barrier dysfunction, and systemic metabolic disorders. These interacting factors converge on the gut microbiota, creating a vicious cycle that increases susceptibility to obesity, MASLD, and other nutrition-related metabolic diseases. Understanding these synergistic interactions is essential for accurate environmental risk assessment, as vulnerable populations may exhibit lower thresholds for MP toxicity. MASLD, metabolic dysfunction-associated steatotic liver disease; MP, microplastic.

**FIGURE 4:**
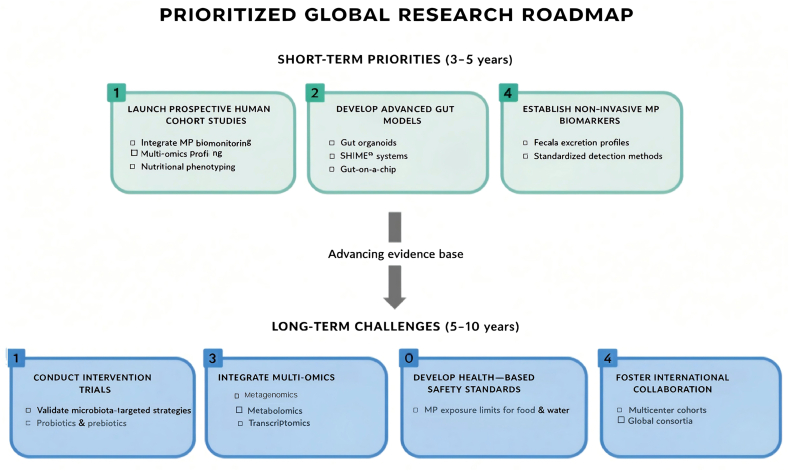
A prioritized global research roadmap. Based on the evidence synthesis in this Review, we propose a staged research agenda to address critical knowledge gaps. Short-term priorities (3–5 y) are defined by their feasibility and potential to establish foundational evidence and include the following: *1*) launching large-scale, prospective human cohort studies integrating robust MP biomonitoring with multiomics profiling; *2*) developing advanced gut models (e.g., gut organoids, SHIME) to elucidate cellular mechanisms; and *3*) establishing standardized, noninvasive biomarkers for MP exposure. Long-term challenges (5–10 y) require sustained interdisciplinary effort and include the following: *1*) conducting intervention trials to validate microbiota-targeted nutritional strategies (e.g., prebiotics, probiotics); *2*) integrating metagenomics, metabolomics, and transcriptomics to uncover novel pathogenic pathways; *3*) developing health-based safety standards for MP in food and water; and *4*) fostering international collaboration through multicenter cohorts and consortia. This roadmap aims to translate mechanistic insights into evidence-based policies and public health interventions. MP, microplastic; SHIME, Simulator of Human Intestinal Microbial Ecosystem.

In summary, we delineate a coherent network of mechanisms whereby MP, through the central node of the gut microbiota, drives a multisystem failure in nutritional homeostasis. This integrated perspective positions the gut microbiota not merely as a victim of MP toxicity but as the pivotal mediator translating environmental exposure into the spectrum of malnutrition disorders. By systematically synthesizing a fragmented body of evidence and integrating it into visual mechanistic frameworks (Figures 2, 3, and 4), this review provides a conceptual model of the “MP–gut microbiota–nutrition” axis. This model serves as a foundational hypothesis, charting the course for future research to validate, refine, or challenge the proposed pathways, particularly in human populations and under real-world exposure scenarios. A critical yet overlooked aspect is that the GLP-1/PYY response to SCFAs may be blunted in obesity, suggesting that MP could further entrench hyperphagia by weakening the very anorexigenic signals designed to counteract overeating. This feed-forward loop remains untested but represents a potentially high-gain pathway linking environmental plastics to the obesity epidemic. The nutritional implication is that MP exposure may create a state of functional food intake dysregulation that is independent of dietary quality. This would complicate behavioral nutrition interventions that rely on intact satiety signaling and suggests that populations with high MP exposure may require adjunctive strategies—such as SCFA-targeted prebiotics—to restore normal appetite control.

### Key mechanisms linking MP to malnutrition via gut microbiota: evidence strength and critical gaps

The 4 mechanisms differ substantially in their evidentiary support, a distinction critical for prioritizing future research.

Established mechanisms with causal validation:

Mechanism 1 (Energy/Macronutrient): Supported by robust evidence from animal models showing MP-induced depletion of SCFA-producing bacteria and altered BA signaling [20,22,41], and causally validated by FMT studies demonstrating that MP-perturbed microbiota transfer recapitulates metabolic defects [47]. Direct human validation is needed.

Mechanism 2 (Barrier/Endotoxemia): Strongly implicated by studies demonstrating MP-induced downregulation of tight junction proteins and increased systemic inflammation in rodents [[51], [52], [53]]; FMT experiments confirm the microbiota mediates barrier defects [48]. Its contribution to human “leaky gut” syndromes remains an open question.

Mechanism with partial behavioral evidence:

Mechanism 4 (Appetite/Gut–Brain Axis): Suggested by MP-altered feeding behavior and hormone secretion in mice [61,63], yet its translational relevance to human eating behavior awaits confirmation.

Hypothesis-generating mechanism requiring in vivo validation:

Mechanism 3 (Micronutrient Bioavailability): Currently lacks direct in vivo evidence, relying on in vitro adsorption data [60] and theoretical links to dysbiosis-impaired vitamin synthesis. Labeled this pathway explicitly as a hypothesis for risk assessment purposes until validated.

Cross-cutting limitation: the mechanistic evidence reviewed above derives predominantly from studies using MP doses (0.1–100 mg/kg/d in rodents) that exceed estimated human environmental exposure (0.1–5 μg/kg/day) by 10^3^- to 10^6^-fold [3,5,20,22,41]. Although some studies have begun to employ environmentally relevant doses (0.01–1 mg/d in mice) [50] and have detected significant biological effects-1, whether the full spectrum of pathways described in this review operates at environmentally relevant concentrations remains the central unanswered question for human risk assessment.

## Amplifying Effects: Synergistic Interactions between Microplastics and Dietary Risk Factors

The synergistic interactions between MP and dietary or developmental factors are conceptualized in Figure 3, whereas a prioritized global research roadmap is presented in Figure 4. The deleterious impacts of MPs on the gut microbiota and host nutrition do not occur in isolation. They converge with other global challenges, such as pandemics, which can exacerbate the double burden of malnutrition through complex interactions with the gut microbiota [10]. Understanding these synergistic interactions is paramount for accurately assessing the real-world public health threat posed by MP.

### Synergy with the WSD and immune dysregulation

The WSD, characterized by high amounts of fats, sugars, and ultraprocessed foods, is a well-established driver of gut dysbiosis and low-grade chronic inflammation. Compelling evidence indicates that MPs and WSD can act synergistically to worsen metabolic disturbances. For instance, mice fed a high-fat diet and exposed to PS MP exhibited more severe intestinal barrier dysfunction, systemic inflammation, and glucose intolerance compared with those on a normal diet [30]. This synergy likely originates from the baseline dysbiosis induced by WSD, such as an elevated Firmicutes/Bacteroidetes ratio and reduced abundance of SCFA-producing bacteria, which renders the gut ecosystem more susceptible to MP-induced perturbations [26,56]. The concomitant increase in intestinal permeability from both insults facilitates a vicious cycle of metabolic endotoxemia, whereby MPs may exacerbate the translocation of LPS and amplify chronic inflammation, thereby promoting the progression of conditions such as MASLD [26,56].

This MP-diet synergy may extend to immune homeostasis, although direct evidence linking MP to food allergy in humans is currently lacking.

### Programming effects of early-life exposure

Early life, including the prenatal and infant periods, represents a critically vulnerable window for gut microbiota assembly and immune and metabolic programming, as underscored by the Developmental Origins of Health and Disease (DOHaD) hypothesis. The detection of MPs in placental tissue confirms the reality of early-life exposure [2]. Animal and in vitro models suggest that such exposure can have long-lasting consequences. Maternal MP exposure can alter the initial gut colonization of offspring, and an infant gut model showed that polyethylene MP exposure disturbed the immature gut microbiota [57]. Although these findings derive from an in vitro model, they underscore the plausibility of early-life susceptibility. A pilot study in preschool children further reported a correlation between MP exposure and gut microbiota alterations [5]. Because of physiological immaturity, including higher intestinal permeability and a developing immune system, coupled with frequent hand-to-mouth behavior, infants and young children are particularly susceptible. Early-life MP exposure may thereby program the microbiota-immune-metabolism axis, potentially increasing risk of obesity, diabetes, and other nutrition-related metabolic diseases later in life [2,57], constituting a pressing intergenerational public health issue. The interplay between MPs, diet, and early-life exposure underscores a complex public health challenge, necessitating a prioritized and collaborative research agenda to translate mechanistic insights into actionable solutions. This integrated perspective on synergistic amplification and the corresponding research priorities is visually synthesized in Figures 3 and 4, which together serve as a roadmap for future investigations and policy development.

## Research Gaps and Future Directions

Despite compelling evidence from preclinical models linking MP exposure to gut microbiota disruption and subsequent nutritional impairments, critical knowledge gaps hinder a definitive understanding of their public health impact. This section outlines key research limitations and proposes a prioritized agenda to advance the field.

### Establishing causal links in human populations

Current evidence is largely derived from animal models (e.g., mice, zebrafish) and in vitro human gut simulations [20,22,23,39,49,66]. Although these studies consistently demonstrate MP-induced dysbiosis and metabolic dysfunction, human epidemiological data remain sparse and correlative. All human evidence published to date, including studies in preschool children [5], university students [7], and young adults with high take-out food consumption [2], derives exclusively from cross-sectional designs that cannot establish temporality or causality. These studies have assessed MP exposure through fecal concentrations and dietary questionnaires rather than direct blood or tissue measurements, further limiting causal inference. No prospective cohort study has yet been conducted that integrates validated MP biomonitoring with nutritional phenotyping and gut microbiota profiling. This represents the single most critical gap in translating the MP–gut microbiota–nutrition axis from preclinical models to human health. For instance, a pilot study in preschool children reported an association between MP exposure and altered gut microbiota but could not infer causality [57]. Addressing this gap requires well-designed longitudinal cohort studies that integrate robust MP biomonitoring, deep phenotypic nutrition assessment, and multiomics profiling [2]. Such efforts should prioritize populations with high environmental MP exposure and varied nutritional status to elucidate dose–response relationships and temporal dynamics. An efficient alternative to launching entirely new cohorts would be to nest MP exposure assessment within ongoing large-scale nutritional epidemiology studies (e.g., the ALSPAC, Generation R, or Nutrition Transition cohorts), leveraging their existing infrastructure for dietary assessment, anthropometric monitoring, and biospecimen banking to enable retrospective and prospective analyses of MP–nutrition associations at marginal incremental cost.

### Advancing mechanistic understanding with realistic exposure models

Human exposure to MP is characterized by chronic, low-dose mixtures of varied polymer types, sizes, and shapes, which are rarely replicated in current experimental designs [5,6,67]. The dose discrepancy between experimental and environmental exposure—often 10^3^- to 10^6^-fold—represents a fundamental challenge for the translatability of mechanistic findings. Moreover, environmental aging (e.g., UV degradation, biofilm colonization) alters MP physicochemical properties and toxicity, as demonstrated in soil fauna studies [45,68]. Host factors—such as baseline microbiota composition, dietary patterns, genetic background, and life stage—further modulate individual susceptibility [[55], [56], [57],^69^]. Future studies must incorporate environmentally relevant MP concentrations—estimated to be in the micrograms per kilogram per day range for dietary exposure [3,5]—and employ chronic exposure durations that better reflect the lifelong nature of human MP ingestion. When higher doses are necessary for mechanistic interrogation, studies should include dose–response arms that span from environmentally relevant to high-exposure levels to enable threshold identification and risk characterization. The development of physiologically based toxicokinetic models may further improve cross-species dose extrapolation and inform the design of human-relevant exposure experiments.

Future studies must incorporate environmentally relevant MP mixtures and account for host variability to improve translational relevance. Moreover, the near-exclusive reliance on pristine spherical particles in current experimental designs represents a critical exposure-relevance gap. Future mechanistic studies should adopt environmentally aged MP models, generated through standardized UV irradiation, mechanical abrasion, or environmental incubation protocols that recapitulate surface oxidation, fragmentation into irregular morphologies, and biofilm colonization [7,59]. Head-to-head comparisons of pristine compared with aged particles within the same experimental system should be prioritized to quantify the contribution of aging-dependent physicochemical changes to gut microbiota perturbation, barrier dysfunction, and micronutrient adsorption. Advanced experimental models, including germ-free animals, FMT studies [22,57], and human-relevant in vitro systems (e.g., gut-on-a-chip, organoids, SHIME) [42,52], are essential to strengthen mechanistic causality and elucidate cellular interactions. For the micronutrient pathway specifically, investigations employing physiologically relevant MP concentrations in advanced gut models with integrated micronutrient absorption assessment are urgently needed to determine whether this pathway contributes to human “hidden hunger,” particularly in populations already at risk [17,20,60]. Emerging analytical approaches—including machine learning-based integration of multiomics data (metagenomics, metabolomics, and transcriptomics) and advanced in vitro models such as gut-on-a-chip with integrated immune components—offer unprecedented opportunities to dissect causal pathways linking MP exposure to nutritional outcomes.

### Overcoming technical barriers for risk assessment

Accurate quantification of internal MP exposure is foundational yet hampered by methodological limitations. Current techniques (e.g., pyrolysis-GC/MS, Raman microscopy) struggle to detect nanoplastics in complex biological matrices and are prone to contamination artifacts [3,70]. Developing standardized, sensitive, and noninvasive biomarkers, such as fecal excretion profiles of MP and their additives, is urgently needed to enable large-scale human biomonitoring [70,71].

Given the multifaceted nature of MP exposure and its potential health impacts, a staged and prioritized research agenda is essential. In the short term (3–5 y), the highest priority must be given to establishing causal links in human populations through prospective cohort studies integrating robust MP biomonitoring with multiomics profiling [2,5]. Concurrently, advanced experimental models must be deployed to strengthen mechanistic causality.

Addressing long-term challenges (5–10 y) demands sustained, concerted effort across several frontiers. Achieving systems-level insight through the integration of metagenomics, metabolomics, and transcriptomics will be crucial to uncover novel pathogenic pathways [22,[40], [41], [42],^72^]. Preclinical evidence suggests that specific probiotics can bind MP and repair gut environments, and prebiotics might help restore microbial homeostasis [[73], [74], [75]]; these microbiota-targeted interventions, already validated in clinical trials for childhood malnutrition [76,77], should be evaluated in the context of MP exposure. Ultimately, this research must inform policy-relevant risk assessment by supporting the development of health-based exposure limits for MP in food and water [1,70]. Success hinges on fostering international collaboration through multicenter cohorts and consortia to capture diverse exposure patterns and host responses, thereby accelerating the translation of evidence into planetary health policies.

## Conclusions and Public Health Implications

In conclusion, this review establishes that the journey of MPs does not end in the environment; rather, ingested MPs have the capacity to disrupt nutritional homeostasis, as demonstrated in preclinical models. We have consolidated evidence supporting the concept of an “MP–gut microbiota–nutrition” axis and propose it as a mechanistic hypothesis that requires targeted evaluation under environmentally realistic human exposure conditions. This framework challenges conventional silos of environmental science and nutrition, arguing that planetary health and human nutritional well-being are inextricably linked.

Of the 4 pathways, 2—impaired energy metabolism and gut barrier dysfunction—are firmly grounded in preclinical evidence and have received causal support from FMT experiments [22,48]; the micronutrient bioavailability pathway, by contrast, remains an important but untested hypothesis. Critically, all human evidence to date is cross-sectional and cannot establish causality; the field now urgently requires prospective cohort studies with integrated MP biomonitoring, nutritional phenotyping, and gut microbiota profiling to determine whether the mechanistic pathways delineated here operate at environmentally relevant exposure levels in human populations. This framework, although compelling, requires targeted evaluation under environmentally realistic human exposure conditions before causal links to human malnutrition can be established.

### Policy-level interventions: source control and regulatory action

The recent convergence of international initiatives provides a policy window for addressing MP pollution within nutritional governance. The FAO has identified MP contamination as an emerging food safety concern, publishing a review on dietary MP exposure in 2022 and launching a dedicated report on microplastic pollution in agricultural soils in 2025, together with a forthcoming “Plastics, Agri-food Systems, and One Health Framework” anticipated in 2026. In parallel, the WHO has systematically assessed the potential health risks of dietary MP exposure through its 2019 report on microplastics in drinking water and its 2022 comprehensive assessment of dietary and inhalation exposure to nano- and MP particles. These assessments consistently identify the lack of standardized analytical methods and the absence of human health-based exposure thresholds as critical barriers to regulatory action. Building on this scientific groundwork, we recommend the following coordinated policy actions.

Source reduction through robust plastic lifecycle governance is foundational. Enforced extended producer responsibility schemes can incentivize safer, biodegradable alternatives [1,3]. Concurrently, developing health-based safety standards for MP in food and water is a critical priority, requiring targeted risk assessments to establish science-based guidance values [70]. The Codex Alimentarius Commission, as the joint FAO/WHO food standards program, provides the natural institutional home for developing such standards, building upon the scientific assessments already conducted by both organizations. Additionally, national governments should leverage the ongoing Intergovernmental Negotiating Committee process, facilitated by the United Nations Environment Programme, to ensure that the future global plastics treaty explicitly recognizes the nutritional health dimensions of plastic pollution and mandates the integration of MP monitoring into existing food safety and nutritional surveillance systems. Such assessments should account for the enhanced toxicity of environmentally aged MP relative to the pristine particles used in most laboratory studies, given that surface oxidation, biofilm formation, and irregular morphology collectively amplify gut-level hazards [27,28]. Public health frameworks must systematically integrate MP exposure assessment into existing food safety and nutritional surveillance systems, including standardized protocols for biomonitoring in vulnerable populations (e.g., children, pregnant women) and incorporation of exposure data into national health databases.

### Toward future clinical and nutritional translation

Embedding MP risk in clinical assessment represents a paradigm shift toward more holistic care. Should future human studies confirm causal relationships and establish dose–response metrics, MP exposure history could inform risk stratification for metabolic disorders such as obesity and MASLD [26,55,56], particularly in individuals consuming WSD. Preclinical evidence suggests that probiotics can bind MP and repair gut environments, and prebiotics may help restore microbial homeostasis [[73], [74], [75],^78^]; these microbiota-targeted interventions, already validated for childhood malnutrition [76,77], warrant evaluation in populations with documented MP exposure.

Clinical populations at potential risk including patients with pre-existing gut barrier dysfunction (e.g., inflammatory bowel disease), those receiving enteral nutrition via plastic tubing, and individuals with high dietary exposure to ultraprocessed foods may represent vulnerable subgroups warranting clinical awareness. Although direct evidence is lacking, clinicians should consider MP exposure as a potential environmental modifier in patients with unexplained metabolic disturbances or refractory micronutrient deficiencies. Pending confirmatory human studies, clinicians may consider adopting a precautionary approach in vulnerable populations by the following: *1*) eliciting dietary exposure to ultraprocessed and plastic-packaged foods as part of nutritional assessment; *2*) prioritizing gut barrier-supporting interventions (e.g., adequate dietary fiber, zinc) in patients with suspected high MP exposure; and *3*) advocating for the use of non-plastic materials for enteral nutrition delivery in high-risk infants and children.

### Public engagement: awareness and behavioral change

Effective public communication and community engagement are essential to drive exposure reduction and collective action. Translating complex evidence on MP sources, exposure routes, and gut-mediated health risks into accessible messaging fosters informed decision-making without inducing undue alarm. Promoting plastic-reduction lifestyles, such as encouraging reusable containers, minimally packaged foods, and alternatives to single-use plastics, is crucial to lower personal MP exposure at the household level [32,36].

### Toward an integrated nutritional risk assessment framework

A comprehensive understanding of malnutrition requires integrating environmental determinants like MPs. Future nutritional guidelines should incorporate an environmental risk dimension, systematically evaluating MP exposure through improved dietary surveys and validated noninvasive biomarkers. This would enable population stratification based on combined risks from MPs, WSD, and pre-existing gut conditions, paving the way for more personalized and effective nutritional interventions. Achieving this vision, however, first requires addressing the fundamental research gaps outlined earlier under “Research Gaps and Future Directions.”

### A call for interdisciplinary action

The pervasive nature of plastic pollution and its potential impact on nutritional health through the gut microbiota demand nothing less than a paradigm shift in our approach to planetary and public health. Addressing the challenge posed by the “MP–gut microbiota–nutrition” axis requires coordinated efforts across environmental science, nutrition, medicine, policy, and education. Ultimately, protecting human nutritional health in the Plastic Age demands that we reframe plastic pollution not as a distant environmental concern but as a proximate and pervasive determinant of malnutrition, a paradigm that fundamentally connects planetary health to human nutritional well-being.

## Author contributions

The authors’ responsibilities were as follows – HL: data curation, formal analysis, writing – original draft; XY, YP: data curation, methodology, writing—review and editing; GW: conceptualization, visualization, data curation, writing—review and editing; WK: formal analysis, software; CH: supervision, funding acquisition, writing – original draft, writing—review and editing; and all authors: read and approved the final manuscript.

## Ethics statement

This review is based exclusively on previously published scientific literature and did not involve the conduct of any new experiments involving human participants or animals. All original studies cited in this work were conducted in accordance with relevant ethical standards: human studies adhered to the principles of the Declaration of Helsinki, and animal studies followed the ARRIVE guidelines. Ethical approvals were obtained by the respective original investigators for all cited research.

## Data availability

All data supporting the findings of this review are derived from previously published studies cited in the reference list. No new data were generated or analyzed in this work. Further inquiries can be directed to the corresponding author.

## Funding

This work was supported by Sanming Project of Medicine in Shenzhen（No. SZSM202311021), Research Initiation Fund of Longgang District Maternity and Child Healthcare Hospital (Y2024011), and Key Medical Disciplines in Longgang District.

## Declaration of generative AI and AI-assisted technologies in the writing process

As part of our manuscript preparation process, the authors acknowledge the use of DeepSeek, a generative AI tool, to assist in the literature review process and in refining certain textual elements of this manuscript. Specifically, DeepSeek was utilized to support the initial screening and summarization of a subset of relevant publications, and to aid in the linguistic polishing and clarity of expression in several sections. All AI-generated content or suggestions were critically reviewed, verified, and substantively edited by the authors, who assume full responsibility for the accuracy, integrity, and scientific validity of the final work. This use of AI tools aligns with the journal's policy on transparent reporting of manuscript preparation assistance.

## Conflict of interest

The authors report no conflicts of interest.
