# Supplementary material for: The Microplastic–Gut Microbiota Axis: A Unifying Framework Linking Environmental Pollution to Undernutrition, Obesity, and Metabolic Dysfunction
Source: Curr Dev Nutr. 2026 Jul 21;10(8):109445. doi: 10.1016/j.cdnut.2026.109445 (PMC13485666; doi:10.1016/j.cdnut.2026.109445)
Supplement: Supplementary file 1 [file mmc2.pdf]

PRISMA 2020 flow diagram for new systematic reviews which included searches of databases and registers only

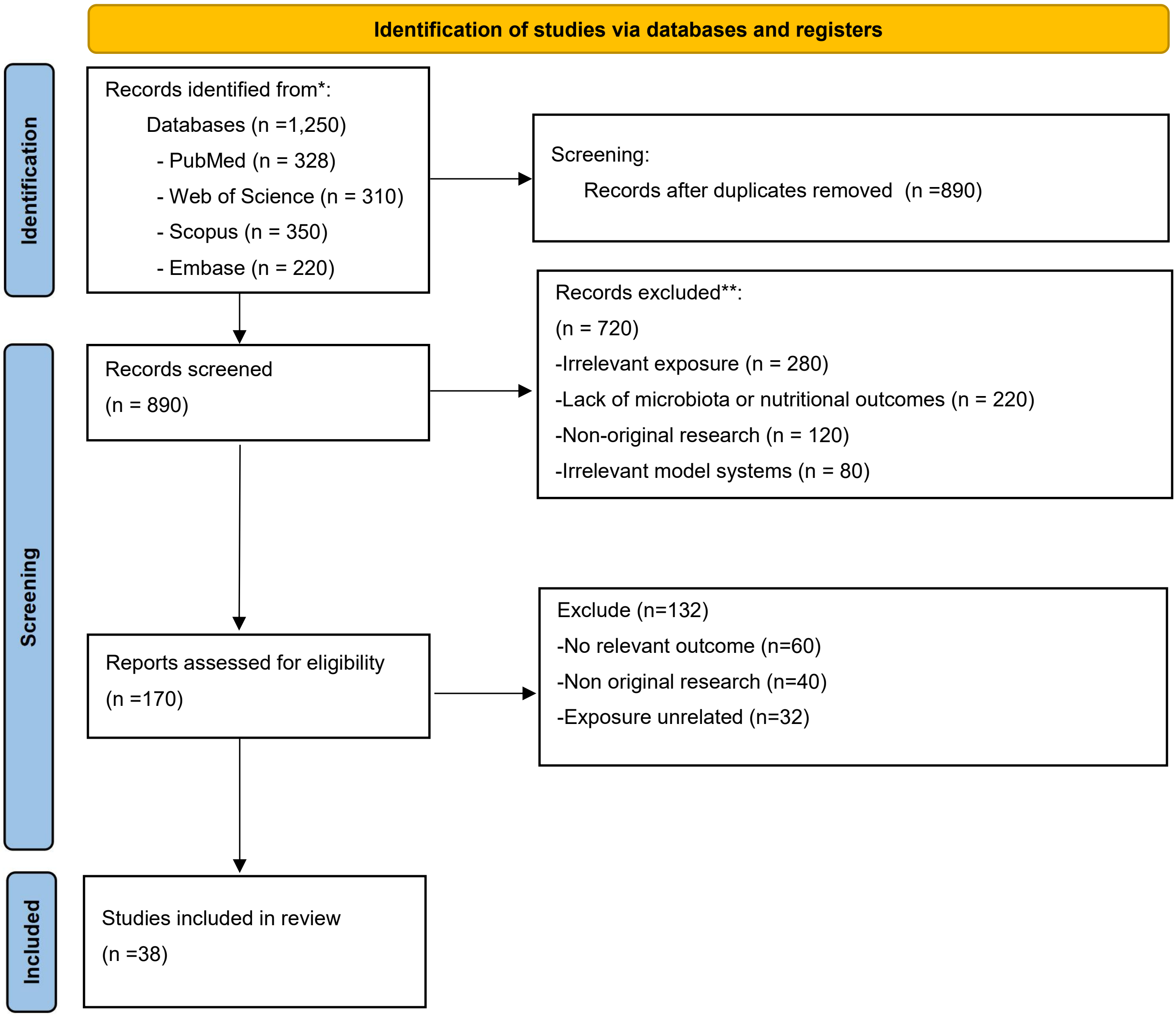

*Consider, if feasible to do so, reporting the number of records identified from each database or register searched (rather than the total number across all databases/registers).

**If automation tools were used, indicate how many records were excluded by a human and how many were excluded by automation tools.

Source: Page MJ, et al. BMJ 2021;372:n71. doi: 10.1136/bmj.n71.

This work is licensed under CC BY 4.0. To view a copy of this license, visit https://creativecommons.org/licenses/by/4.0/

Supplementary Figure 1 | PRISMA 2020 flow diagram illustrating the literature search and study selection process. The systematic search across five databases (PubMed, Web of Science, Scopus, Embase, Cochrane) yielded 1,250 records. After removing duplicates, 890 records underwent title/abstract screening, with 720 excluded for irrelevance (e.g., non-oral exposure, lack of microbiota/nutrition outcomes, or non-original articles). Of the 170 full-text articles assessed for eligibility, 132 were excluded with reasons (e.g., no relevant outcomes, inappropriate exposure, or non-original research), resulting in 38 studies included in the final systematic review.
