# Supplementary material for: The Microplastic–Gut Microbiota Axis: A Unifying Framework Linking Environmental Pollution to Undernutrition, Obesity, and Metabolic Dysfunction
Source: Curr Dev Nutr. 2026 Jul 21;10(8):109445. doi: 10.1016/j.cdnut.2026.109445 (PMC13485666; doi:10.1016/j.cdnut.2026.109445)
Supplement: Supplementary file 2 [file mmc1.docx]

Supplementary Information

Supplementary Information: Detailed Methodology

Appendix S1: Comprehensive Literature Search Strategy

This appendix presents the complete search strategy used for this systematic review, conducted in accordance with PRISMA guidelines across five major electronic databases from inception through October 31, 2025.

1. PRISMA 2020 Checklist

The PRISMA 2020 checklist for this review is provided in Supplementary Table 1.

2. Literature Search Strategy

A comprehensive literature search was conducted in PubMed, Web of Science, and Scopus from inception through October 31, 2025. The search combined terms related to four thematic areas: (1) microplastics (e.g., microplastic, nanoplastic, polystyrene, polyethylene, polyvinyl chloride); (2) gut microbiota (e.g., gut microbiome, gut microbiota, intestinal flora, intestinal microbiome); (3) nutrition and metabolism (e.g., malnutrition, undernutrition, obesity, metabolic syndrome, short-chain fatty acid, bile acid, Western diet); and (4) mechanistic pathways (e.g., gut barrier, intestinal barrier, tight junction, endotoxemia, lipopolysaccharide, gut–brain axis, appetite regulation, inflammation). Detailed search strings are available from the corresponding author upon request.

No restrictions were placed on study design or population. Non-English publications and studies involving non-oral exposure routes (inhalation, dermal, injection) were excluded during manual screening.

3. Study Selection Process

- Platform: Rayyan (QCRI, https://www.rayyan.ai/)

- Screening: Two independent reviewers (XY, JC) screened titles/abstracts against predefined criteria

- Full-text review: Conducted independently by same reviewers

- Conflict resolution: Discrepancies resolved through discussion or consultation with third reviewer (GW)

- Selection summary: See PRISMA flow diagram (Supplementary Figure 1)

4. Inclusion and Exclusion Criteria

| Criteria | Inclusion | Exclusion |
| --- | --- | --- |
| Population/Model | Any (humans, animals, in vitro systems) | – |
| Exposure | Oral exposure to microplastics or nanoplastics | Non-oral exposures (inhalation, dermal, injection) |
| Outcomes | Gut microbiota composition/function AND/OR nutritional/metabolic parameters | No relevant outcomes |
| Publication type | Original research articles | Reviews, editorials, conference abstracts, commentaries |
| Language | English | Non-English |
| Date | Inception to October 31, 2025 | After October 31, 2025 |

5. Data Extraction Items

A standardized data extraction form captured:

- Study characteristics: Authors, year, country, study design

- Model details: Species/strain, sample size, sex, age

- MP properties: Polymer type, size, shape, concentration/dose, exposure duration, presence of additives

- Microbiota analysis: Method (16S rRNA sequencing, metagenomics, etc.), targeted region, bioinformatics pipeline

- Key outcomes: α/β-diversity metrics, taxonomic changes, SCFA concentrations, barrier function markers (tight junction proteins, permeability), inflammatory markers (LPS, cytokines), metabolic parameters (glucose, insulin, lipids), nutritional status

6. Quality Assessment Tools

| Study Type | Assessment Tool | Key Domains |
| --- | --- | --- |
| Animal studies | SYRCLE's RoB tool | Selection bias, performance bias, detection bias, attrition bias, reporting bias |
| In vitro studies | CREDIBLE checklist | Study design, model system characteristics, exposure characterization, outcome assessment, reporting quality |
| Observational studies | NIH Quality Assessment Tool | Study population, exposure assessment, outcome measures, confounding, follow-up |

Studies were not excluded based on quality; assessments informed interpretation and discussion of findings.

7. Evidence Synthesis Approach

Due to substantial heterogeneity in exposure parameters (polymer type, size, dose, duration) and outcome measures, meta-analysis was deemed inappropriate. A narrative synthesis was conducted by:

1. Categorizing evidence by mechanistic theme (energy metabolism, barrier function, micronutrient bioavailability, gut–brain axis) and study model (rodent, zebrafish, in vitro human gut)

2. Identifying consistent patterns of MP-induced microbiota disruption and host metabolic perturbations across studies

3. Critically evaluating variations in experimental design and exposure parameters to assess robustness and reproducibility of findings

4. Constructing a unified conceptual framework integrating evidence into the proposed "MPs–gut microbiota–nutrition" axis

8. Supplementary Table 1. Characteristics of Included Studies

| Reference | Study Design | Model/Subjects | MP Characteristics (polymer, size, dose, duration) | Microbiota Findings | Nutritional/Metabolic Outcomes |
| --- | --- | --- | --- | --- | --- |
| Shi et al., 2024 [5] | Meta-analysis | Aquatic animals (multiple studies) | Various polymers, sizes, doses | MPs predominantly alter β-diversity rather than α-diversity \| | N/A (meta-analysis) |
| Qiu et al., 2025 [7] | Human cross-sectional study | University students | MPs from take-out containers (various polymers) | Diet-driven gut dysbiosis; altered α-diversity | N/A |
| Zhang et al., 2024 [20] | Animal study | Male C57BL/6 mice | Polystyrene (PS); 0.5, 5, 50 μm; 0.1, 1, 10 mg/kg/day for 28 days | ↓ α-diversity; ↓ Lachnospiraceae, Ruminococcaceae; ↑ Proteobacteria | Multi-organ damage; altered fecal metabolites |
| Lu et al., 2018 [21] | Animal study | Male ICR mice | PS; 5 μm; 0.1, 1, 10 mg/L in drinking water for 5 weeks | ↓ α-diversity; altered β-diversity; ↓ Bacteroidetes, ↑ Firmicutes | Hepatic lipid metabolism disorder; ↑ total cholesterol, triglycerides |
| Jin et al., 2018 [22] | Animal study | Adult zebrafish | PS; 5, 20, 50 μm; 0.1, 1, 10 mg/L for 14 days | ↓ α-diversity; ↓ Bacteroidetes, ↑ Proteobacteria; ↓ SCFA-producing bacteria | Intestinal inflammation; ↑ TNF-α, IL-1β |
| Chen et al., 2022 [23] | Animal study | Male BALB/c mice | Polyvinyl chloride (PVC); 1–10 μm; 0.1, 1, 10 mg/kg/day for 42 days | ↓ α-diversity; ↓ Firmicutes, ↑ Bacteroidetes; ↓ SCFA producers | Liver injury; ↑ ALT, AST; gut barrier dysfunction (↓ ZO-1, occludin) |
| Xu et al., 2024 [24] | Animal study | Male C57BL/6 mice | Polyethylene (PE); 10–100 μm; 0.1, 1, 10 mg/kg/day for 35 days | ↓ α-diversity; altered β-diversity; ↓ Lactobacillus, ↑ Helicobacter | Liver injury via TLR2/NF-κB/NLRP3 pathway |
| Huang et al., 2023 [25] | Animal study | Male C57BL/6 mice | PS; 5 μm; 0.1, 1, 10 mg/L in drinking water for 8 weeks | ↓ α-diversity; ↑ Firmicutes/Bacteroidetes ratio; ↓ SCFA producers | Adiposity; ↑ body weight gain, fat mass; ↑ fatty acid synthesis genes |
| Kou et al., 2025 [26] | Animal study | Male C57BL/6 mice | PS; 0.5, 5 μm; 0.1, 1, 10 mg/kg/day for 6 weeks | ↓ α-diversity; altered β-diversity; ↓ SCFA producers | Impaired brown/beige adipocyte function; ↓ UCP1, PGC-1α |
| Cui et al., 2025 [27] | Animal study | Male C57BL/6 mice | Pristine vs. UV-aged PE; 0.01–1 mg/day; dietary exposure | Aged MPs enriched Akkermansia, Parasutterella, Turicibacter; reduced Lactobacillus | Hepatic dysfunction; elevated reactive oxygen species in small intestine and liver |
| Jia et al., 2024 [28] | Animal study | Male C57BL/6 mice | Pristine vs. UV-aged PS nanoplastics; specific concentration and duration as per cited study | N/A (study focused on oxidative stress markers rather than microbiota per se) | Aggravated intestinal barrier damage; overproduction of reactive oxygen species and hydroxyl radicals |
| Okamura et al., 2023 [29] | Animal study | Male C57BL/6 mice | PS; 1–10 μm; 0.1, 1, 10 mg/kg/day with normal or high-fat diet for 12 weeks | ↓ α-diversity; ↓ Lachnospiraceae, Ruminococcaceae; ↑ Proteobacteria | Exacerbated metabolic outcomes on high-fat diet; ↑ insulin resistance |
| Jiang et al., 2021 [30] | Animal study | Male C57BL/6 mice | PS; 5 μm; 1 mg/kg/day + tributyltin (TBT) for 8 weeks | ↓ α-diversity; altered β-diversity; ↓ SCFA producers | Bile acid dysregulation; altered FXR-FGF15 signaling |
| Cox et al., 2019 [31] | Human exposure assessment | Humans (dietary intake) | N/A (review of human consumption) | N/A | Estimated human MP intake |
| Ke et al., 2023 [32] | Human pilot study | Preschool children (n=36) | Fecal MPs detected (various polymers) | Association between MP exposure and gut microbiota alterations; ↓ α-diversity | N/A |
| Li et al., 2020 [33] | Animal study | Male ICR mice | PE; 10–100 μm; 0.1, 1, 10 mg/L in drinking water for 5 weeks | ↓ α-diversity; altered β-diversity; ↓ Bacteroidetes, ↑ Firmicutes | Inflammation; ↑ IL-6, TNF-α in colon |
| Chen et al., 2025 [34] | Animal study | Male Sprague-Dawley rats | Nonphagocytosable MPs; 10–100 μm; 1 mg/kg/day for 28 days | Bile acid–gut microbiota interactions disrupted | Colon inflammatory injury; ↓ tight junctions |
| Huang et al., 2023 [35] | Animal study | Male Sprague-Dawley rats | PS; 50 nm, 5 μm; 0.1, 1, 10 mg/kg/day for 28 days | ↓ α-diversity; altered β-diversity; ↓ SCFA producers | Gut barrier dysfunction mediated by exosomal miRNAs |
| Huang et al., 2021 [36] | Review | N/A | N/A | N/A | N/A |
| Chen et al., 2022 [37] | Animal study | Male BALB/c mice | PVC; 1–10 μm; 0.1, 1, 10 mg/kg/day for 42 days | ↓ α-diversity; altered β-diversity; ↓ SCFA producers | Gut barrier dysfunction, microbiota dysbiosis, metabolic disorder |
| Chen et al., 2025 [38] | Review | Crustaceans | Various | N/A | N/A |
| Wang et al., 2024 [39] | Animal study | Male C57BL/6 mice | PS; 5 μm; 0.1, 1, 10 mg/kg/day for 8 weeks | ↓ α-diversity; altered β-diversity; ↓ SCFA producers | Reduced sociality; ↓ brain oxytocin via gut–brain axis |
| Feng et al., 2025 [40] | Human study | University students | MPs from take-out containers | Diet-driven gut dysbiosis; ↓ α-diversity | \| N/A |
| Ding et al., 2022 [41] | Review | Soil fauna | N/A | \|N/A | N/A |
| Li et al., 2021 [42] | Animal study | Freshwater clams (Corbicula fluminea) | PS; 1 μm; 0.1, 1, 10 mg/L for 14 days | N/A | Genotoxicity, intestinal damage |
| Han et al., 2025 [43] | Animal study | Zebrafish | Fibrous MPs; 10–100 μm; 0.1, 1, 10 mg/L for 21 days | N/A | Enhanced toxicity of decabromodiphenyl ethane |
| Deng et al., 2020 [44] | Animal study | Male ICR mice | PE; 10–100 μm; 0.1, 1, 10 mg/L in drinking water for 5 weeks | ↓ α-diversity; altered β-diversity; ↓ SCFA producers | Phthalate release; aggravated gut effects |
| Zhang et al., 2024 [45] | Human quasi-experimental study | University students | Thermal exposure to disposable plastic tableware | Altered gut microbiota and metabolome | N/A |
| Gong et al., 2022 [46] | Meta-analysis | Humans (obesity) | N/A | Firmicutes/Bacteroidetes ratio altered in obesity | N/A |
| Wang et al., 2022 [47] | Animal study | Greater wax moth larvae | PS; <100 μm; dietary exposure | Gut microbiota independent degradation | N/A |
| Wang et al., 2024 [50] | Animal study | Yellow mealworms | Aged PE and PS; <150 μm; dietary exposure for 30 days | Gut microbiota transition; enrichment of plastic-degrading bacteria | N/A |
| Quan et al., 2023 [51] | Animal study | Superworms (Zophobas atratus) | PS; <150 μm; dietary exposure for 30 days | Gut microbiota transition; putative metabolic pathways | N/A |
| Zhang et al., 2024 [48] | In vitro | Human gut microbiome | PET; <100 μm; anaerobic digestion | Identification of PET hydrolytic enzyme | N/A |
| Tamargo et al., 2022 [52] | In vitro | Human gut microbiota (simulated digestion) | PET; 10–100 μm; 0.1, 1, 10 mg/L | Altered bacterial composition and function | Plausible PET biodegradation during digestion |
| Yan et al., 2022 [53] | In vitro | Human fecal microbiota | Phthalates released from MPs; 0.1–100 μg/mL | Inhibited microbial metabolic activity; differential effects on luminal vs mucosal microbiota | N/A |
| Liu et al., 2023 [54] | Field study | Golden pompano aquaculture | MPs from environment | Colonized-microbiota on MPs correlated with intestinal microflora | N/A |
| Ding et al., 2024 [55] | Animal study | Soil fauna (earthworms) | Aged MPs in soil | Aging increases disturbance to gut microbiota | N/A |
| Morrison & Preston, 2016 [56] | Review | N/A | N/A | N/A | SCFA impact on metabolism |
| Huang et al., 2020 [57] | Animal study | Juvenile guppy (Poecilia reticulata) | PE; 10–100 μm; 0.1, 1, 10 mg/L for 21 days | ↓ α-diversity; altered β-diversity; ↓ SCFA producers | Impaired digestive performance; immune response |
| Wang et al., 2021 [58] | Animal study | Honey bees (Apis mellifera) | PS; 5 μm; 0.1, 1, 10 mg/L in sucrose solution for 10 days | Gut microbiota protects against PS toxicity | N/A |
| Fournier et al., 2023 [49] | In vitro | Human gut model (adult) | Various MPs; 10–100 μm; 0.1–10 mg/L | First evidence of MP fate in human digestive tract | N/A |
| Godoy et al., 2019 [59] | Review | N/A | N/A | N/A | MPs as carriers of metals |
| Choi et al., 2024 [61] | Animal study | Adult zebrafish | UV-weathered polypropylene (PP) microplastics; specific concentration and duration as per cited study | Gut microbiota composition altered; UV-weathering amplified microbiome disruption | Neurobehavioral alterations; enhanced toxicity compared to pristine counterparts |
| Psichas et al., 2015 [62] | Animal study | Rodents | N/A | N/A | Propionate stimulates GLP-1 and PYY secretion |
| Yan et al., 2021 [63] | Field study | Shrimp | MPs from environment | Colonized microbiota on MPs correlates with intestinal microbiota | N/A |
| Pabst & Mowat, 2012 [64] | Review | N/A | N/A | N/A | Oral tolerance |
| Wen et al., 2022 [65] | Animal study | Male C57BL/6 mice | PS; 5 μm; 1 mg/kg/day for 6 weeks, then FMT | FMT transferred testicular disorder | N/A |
| Hunt et al., 2024 [66] | Animal study | Mice (DSS colitis) | N/A | SCFA-FFAR2/3-GLP-1 pathway not involved | N/A |
| Chen et al., 2025 [68] | Review | N/A | N/A | N/A | Interaction of pollutants and gut microbiota |
| Fournier et al., 2023 [6] | In vitro | Infant gut model | PE; 10–100 μm; 0.1–10 mg/L | Altered immature gut microbiome | N/A |
| Ma et al., 2025 [69] | Human cross-sectional | Older adults | Fecal MPs detected | Association between MP exposure and gut microbiota/metabolites | N/A |
| Zhao et al., 2024 [70] | Animal study | Zebrafish | Virgin/aged PE; 0.1, 1, 10 mg/L with penthiopyrad for 28 days | Single/combined exposure effects | N/A |
| Wang et al., 2025 [71] | Animal study | Mice | PS; 5 μm; 0.1, 1, 10 mg/kg/day for 6 weeks | Multi-organ crosstalk; transfer toxicity | N/A |
| Li et al., 2024 [72] | Animal study | Earthworms | Freeze-thaw aged MPs | Increased toxicity; enriched pollutant-degrading genera | N/A |
| Yan et al., 2024 [73] | Method development | Humans (fecal samples) | N/A | N/A | Noninvasive method for evaluating MP exposure |
| Zhang et al., 2023 [74] | Animal study | Mice | PS; 5 μm; 0.1, 1, 10 mg/kg/day for 6 weeks | Gut microbiota mediates hepatic injuries via gut–liver axis | N/A |
| Shi et al., 2025 [75] | In vitro / animal | Lactic acid bacteria + mice | PS; 50 nm, 5 μm; 0.1–10 mg/L | LAB reduce MP toxicity through biobinding and gut repair | N/A |
| Kumar et al., 2023 [76] | Animal study | Rats | Spirulina supplementation | Ameliorates iron/protein malnutrition; modulates gut microbiota | Improved growth; reduced oxidative stress |
| Amadieu et al., 2022 [77] | Human pilot | Alcohol use disorder patients | Inulin supplementation | Restored gut microbiota; improved sociability | N/A |
| Chen et al., 2021 [78] | Human trial | Undernourished children | Microbiota-directed food | Promoted weight gain; altered gut microbiota | N/A |
| Barratt et al., 2022 [79] | Human trial | Bangladeshi infants with severe acute malnutrition | Bifidobacterium infantis treatment | Promoted weight gain | N/A |
| Medriano et al., 2025 [60] | Animal study | Adult zebrafish | PE, polyester; environmentally relevant concentrations for 30 days | Altered β-diversity; α-diversity stable; microbiome resilience | N/A |

Note: Only original research articles investigating oral exposure to microplastics/nanoplastics and reporting outcomes related to gut microbiota and/or nutritional status are included. Reviews, meta-analyses, and studies with non-oral exposures are excluded from this table. N/A = not applicable or not reported.
